# MCFANet: a multi-class fusion attention network for motor imagery EEG classification

**DOI:** 10.3389/fnhum.2026.1811759

**Published:** 2026-04-09

**Authors:** Peijie Zhao, Tong Liang, Hao Jia, Azure Dayan, Josep Dinarès-Ferran, Jordi Solé-Casals

**Affiliations:** 1 Data and Signal Processing Group, University of Vic-Central University of Catalonia, Vic, Barcelona, Spain; 2 Faculty of Health Data Science, Juntendo University, Urayasu, Chiba, Japan; 3 School of Medicine, Nankai University, Tianjin, China; 4 Tianjin Key Laboratory of Interventional Brain-Computer Interface and Intelligent Rehabilitation, Nankai University, Tianjin, China; 5 Department of Psychiatry, University of Cambridge, Cambridge, United Kingdom

**Keywords:** attention-based deep learning, brain-computer interface, common spatial pattern, motor imagery EEG, multi-class fusion

## Abstract

**Introduction:**

This paper proposes a Multi-Class Fusion Attention Network (MCFANet) that combines the multi-class spatial filtering outputs of FBCSP with the spatiotemporal feature extraction capability of convolutional neural networks for multi-class motor imagery EEG classification. In multi-class motor imagery decoding, traditional spatial filtering methods extract effective discriminative spatial features but decompose the task into independent binary subproblems, and typically retain only energy statistics while discarding temporal dynamics. Deep learning methods can learn spatiotemporal features but must learn spatial patterns from the beginning, making it difficult to fully capture established neurophysiological priors under limited training samples.

**Methods:**

MCFANet concatenates the spatial filtering outputs from all classes and sub-bands along the channel dimension to construct a virtual channel representation containing the discriminative responses of all classes. The full time series is preserved and fed into a convolutional module for spatiotemporal feature extraction, and a channel attention module adaptively reweights the feature maps to focus on the most discriminative representations. Four-class classification experiments were conducted on two public datasets.

**Results:**

On Dataset 2a, MCFANet achieved an accuracy of 67.94% ±13.70, outperforming FBEEGNet (63.98%) and EEGNet (58.79%). On the High Gamma Dataset, MCFANet achieved 87.10% ±10.09, improving over FBEEGNet by approximately 2.5 percentage points. Paired t-tests and effect size analysis confirm that the improvements over the main baseline methods are statistically significant.

**Discussion:**

The results suggest that reorganizing multi-class spatial discriminative responses into a unified representation that preserves temporal dynamics provides an effective path for bridging traditional spatial filtering and deep learning.

## 1 Introduction

Brain-computer interfaces (BCIs) provide a direct communication link between the brain and external devices such as computers, robots, and vehicles (Wolpaw et al., 2002). Electroencephalography (EEG) is the most commonly used signal acquisition method for non-invasive BCIs, owing to its high temporal resolution and low cost (Wolpaw et al., 2002; Lotte et al., 2018). Motor imagery (MI), which requires subjects to mentally simulate a specific limb movement without actual execution, serves as a stimulus-free BCI paradigm (Pfurtscheller and Neuper, 2001). However, the EEG responses elicited by MI are weaker and less consistent than those produced by real movement (Pfurtscheller and Lopes da Silva, 1999; Neuper et al., 2006; Miller et al., 2010). While binary MI decoding, such as distinguishing left from right hand imagery, has been extensively studied, extending to multi-class scenarios poses additional difficulties: cortical representations of different limbs partially overlap, and inter-subject variability further complicates multi-class discrimination (Brunner et al., 2008; Schirrmeister et al., 2017a).

Traditional MI-EEG classification methods have developed along two main lines. The first is spatial filtering, represented by the Common Spatial Pattern (CSP) algorithm (Ramoser et al., 2000; Koles et al., 1990; Blankertz et al., 2008), which designs spatial filters to maximize the variance difference between classes so as to extract event-related desynchronization/synchronization (ERD/ERS) features induced by MI tasks. Several CSP variants—including Filter Bank CSP (FBCSP) (Ang et al., 2008), Regularized CSP (Lotte and Guan, 2011), and Sparse CSP (Arvaneh et al., 2011)—extend this idea through spectral band selection and regularization. The second line is time-frequency analysis, including wavelet transform (Subasi, 2007), empirical mode decomposition (Park and Chung, 2019), and power spectral density estimation (Pfurtscheller and Lopes da Silva, 1999), which aim to capture multi-scale temporal and spectral features of EEG signals. In addition, Riemannian geometry-based methods map EEG covariance matrices onto the symmetric positive definite (SPD) manifold for classification and have shown notable robustness to noise (Barachant et al., 2012). However, these methods rely on traditional classifiers such as support vector machines (SVM) and linear discriminant analysis (LDA) (Lotte et al., 2018), where feature extraction and classifier training are carried out separately without end-to-end joint optimization. Moreover, CSP and its variants are sensitive to the quality of covariance matrix estimation: under small-sample conditions the covariance estimates become unreliable, degrading the quality of the resulting spatial filters and limiting generalization (Schirrmeister et al., 2017a).

Deep learning offers a path to overcome these bottlenecks. Convolutional neural networks (CNNs) can automatically learn hierarchical spatiotemporal features from raw signals in an end-to-end manner (Lawhern et al., 2018). Early CNN approaches such as DeepConvNet and ShallowConvNet (Schirrmeister et al., 2017a) achieved promising results; the latter mimics the bandpass and spatial filtering pipeline of FBCSP through temporal and spatial convolution layers. However, these networks contain many parameters and tend to overfit on small EEG datasets. EEGNet, proposed by Lawhern et al. (2018), uses depthwise separable convolutions to substantially reduce the parameter count and has become one of the most widely adopted baselines in this field due to its compact design and strong generalization. Despite these advances, purely data-driven networks do not take advantage of the neurophysiological priors underlying MI. Motor imagery produces pronounced ERD/ERS changes in the mu/beta band (8-30 Hz) over the contralateral sensorimotor cortex, and the scalp-level spatial energy distributions differ markedly between, for example, left and right hand imagery—this is precisely the neurophysiological basis that makes CSP effective (Pfurtscheller and Lopes da Silva, 1999). End-to-end networks must learn such patterns from scratch, which is difficult when training samples are limited. To address this, researchers have begun embedding traditional algorithms into network architectures: CSP-Net (Jiang et al., 2024) initializes the spatial convolution weights with CSP filters so that the network starts with effective spatial filtering; FBCNet (Mane et al., 2021) adopts the multi-subband decomposition strategy of FBCSP and extracts spectro-spatial features through filter-bank and variance layers; Graph-CSPNet (Ju and Guan, 2023) constructs graph structures on SPD manifolds in the time-frequency domain and uses Riemannian geometry to capture global geometric relationships in EEG signals.

In deep learning frameworks, attention mechanisms are commonly used to allow networks to assign different levels of importance to different features. The squeeze-and-excitation (SE) module proposed by (Hu et al. 2018) was originally designed for image classification, which assigns a weight to each channel through global pooling. (Woo et al. 2018) extended this idea by adding a spatial attention dimension on top of channel attention. This type of framework has been adopted in EEG-based motor imagery classification. (Altuwaijri et al. 2022) embedded SE blocks into a multi-branch EEGNet and demonstrated its effectiveness on the BCI Competition IV 2a dataset. (Altaheri et al. 2022) proposed ATCNet, which combined multi-head self-attention with a temporal convolutional network to extract features from MI-EEG signals. At the same time, a growing number of studies have explored attention-based designs for MI decoding, including multi-dimensional attention modules tailored to the characteristics of EEG signals (Miao et al., 2023) and the use of channel attention for automatic electrode selection (Tong et al., 2023), lightweight spatial-spectral attention for joint electrode modeling (Han et al., 2025), and a systematic comparative framework for channel attention mechanisms in MI decoding (Wimpff et al., 2024). These attention-based methods improve feature extraction, but their quality remains constrained by the two issues discussed below.

First, discriminative information across multiple classes lacks a unified representation. CSP is inherently a binary method, and its multi-class extension relies on one-vs.-rest decomposition, where each subproblem trains its own spatial filters and classifier in an independent feature space. Because the discriminative responses from different subproblems share no common reference frame, the relative relationships among class-specific responses are difficult to exploit. Hybrid methods such as CSP-Net and FBCNet improve current methods through end-to-end training, but they do not explicitly construct a representation that contains the spatial discriminative responses of all classes. Second, the standard FBCSP pipeline computes log-variance after spatial filtering, reducing an entire time series to a single number. This keeps only the total energy and throws away everything about how that energy changes over time. However, the ERD/ERS elicited by different MI classes differ in onset latency, rate of development, and duration (Pfurtscheller and Lopes da Silva, 1999; Neuper et al., 2006); these temporal differences are themselves informative for classification, yet they are entirely erased by the log-variance computation. Deep learning methods can capture such temporal dynamics, but when applied directly to raw EEG, the network must simultaneously learn spatial filtering and temporal feature extraction from limited data, while CSP already provides a mathematically optimized discriminative spatial projection that is not being utilized.

To address these two issues, this paper proposes the Multi-Class Fusion Attention Network (MCFANet). The main contributions of this work are summarized as follows:

- MCFANet, a fusion attention network for multi-class motor imagery classification. The method concatenates the CSP-filtered outputs from all classes at the input into a virtual channel, allowing the network to process the discriminative responses of all classes within a common feature space simultaneously, rather than solving each binary subproblem independently.
- The full time series after CSP projection is retained instead of being compressed into log-variance scalars as in the standard FBCSP pipeline. This allows the subsequent convolutional layers to learn temporal differences across classes directly from the spatially filtered signals.
- A channel attention module is introduced to adaptively weight the feature maps produced by the convolutional backbone. Since different feature maps contribute differently to classification, the module learns importance weights that guide the network toward the most discriminative representations.
- The method is evaluated on two public datasets with different channel numbers and sample sizes. Paired t-tests and effect size analysis are used to assess statistical significance, and additional analyses including confusion matrices and feature visualization are provided to examine the method in detail.

The remainder of this paper is organized as follows. Section 2 describes the datasets, preprocessing, and the detailed architecture of the proposed method. Section 3 reports the classification results and comparisons with baseline methods. Section 4 discusses the role of key components. Finally, Section 5 concludes the paper.

## 2 Methodology

### 2.1 Dataset description

In this work, classification performance is evaluated on two public datasets, each involving four motor imagery tasks. They are referred to as Dataset 2a and Dataset HGD. Within each dataset, all compared methods were trained and evaluated under identical preprocessing conditions.

The first dataset is BCI Competition IV Dataset 2a (Tangermann et al., 2012), provided by Graz University of Technology. This dataset contains EEG data from 9 subjects. The four motor imagery tasks are left hand, right hand, both feet, and tongue. A total of 25 channels were recorded, of which 22 are EEG channels and 3 are EOG channels. Each subject completed two sessions on different days, with 288 trials per session (72 trials per class). At the beginning of each trial, a fixation cross and an acoustic warning tone were presented. After 2 s, a directional cue indicated the required motor imagery task. Subjects performed the imagery until the fixation cross disappeared at 6 s. The EOG channels are excluded from classification, no additional trial rejection was applied, as the dataset has been widely used in its original form in prior studies (Brunner et al., 2008; Mane et al., 2021). Signals were sampled at 250 Hz and band-pass filtered between 0.5 Hz and 100 Hz. For each trial, a 0-4 s time window is extracted starting from the task cue onset.

High Gamma Dataset(HGD) (Schirrmeister et al., 2017b) contains EEG data from 14 subjects. The four tasks are left hand, right hand, both feet, and rest. Data were recorded from 128 electrodes at a sampling rate of 500 Hz. Similarly, a 0-4 s time window is extracted for each trial. Trials containing artifacts are rejected based on an amplitude threshold. Only 44 channels over the motor cortex area are retained, covering the FC, C, and CP regions along with their surrounding high-density electrodes. The data are then downsampled to 250 Hz. Although this dataset provides an official split into training and test sets, only the official training set is used in the experiments, which is further divided into training and test subsets using a fixed random seed. Each subject has approximately 880 trials.

### 2.2 Multi-class fusion attention network

**Figure 1:**
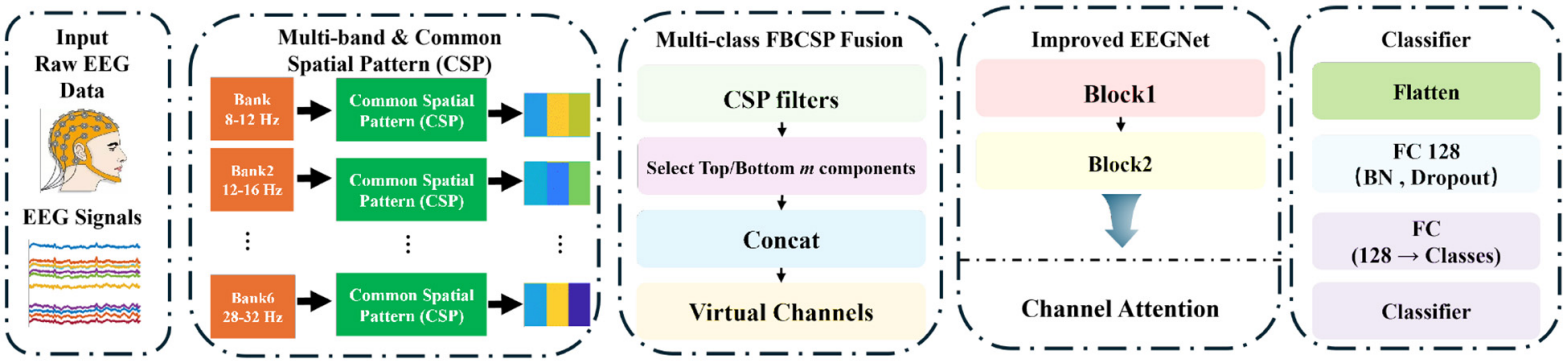
Overall framework of MCFANet. Raw EEG signals are decomposed into multiple sub-bands by a filter bank, and CSP spatial filters are computed within each sub-band. The top *m* and bottom *m* CSP components are selected from each class sub-band pair, and the outputs from all classes and sub-bands are concatenated along the channel dimension to form a virtual channel. The virtual channels are fed into a modified EEGNet, passing through Block 1 (temporal convolution + depthwise spatial convolution), Block 2 (separable convolution), and a channel attention module for feature extraction. The final classification into motor imagery classes is performed by a fully connected layer.

The Multi-Class Fusion Attention Network (MCFANet) combines the multi-band spatial filtering pipeline of FBCSP with the temporal feature extraction capability of EEGNet, and extends both through multi-class filter fusion and channel attention. The method consists of four steps: (1) multi-band decomposition and spatial filtering, (2) multi-class spatial filter fusion, (3) temporal–spatial feature extraction, and (4) channel attention and classification. The overall framework is shown in Figure 1. A detailed layer-by-layer specification of the network is provided in Table 1.

**Table 1:** The model structure of MCFANet.

Layer	Output size	Parameter
Input layer	[*B*, 1, *D, T*]	
BatchNorm2d	[*B*, 1, *D, T*]	
ZeroPad2d	[*B*, 1, *D, T*+63]	(32, 31, 0, 0)
Conv2d	[*B*, 16, *D, T*]	(1, 64)
BatchNorm2d	[*B*, 16, *D, T*]	
Conv2d	[*B*, 32, 1, *T*]	(*D*, 1), *grouped*
BatchNorm2d	[*B*, 32, 1, *T*]	
ELU	[*B*, 32, 1, *T*]	
AvgPool2d	[*B*, 32, 1, *T*//4]	(1, 4)
Dropout	[*B*, 32, 1, *T*//4]	0.5
ZeroPad2d	[*B*, 32, 1, *T*//4+15]	(7, 8, 0, 0)
Conv2d	[*B*, 32, 1, *T*//4]	(1, 16), *grouped*
Conv2d	[*B*, 32, 1, *T*//4]	(1, 1)
BatchNorm2d	[*B*, 32, 1, *T*//4]	
ELU	[*B*, 32, 1, *T*//4]	
AvgPool2d	[*B*, 32, 1, *T*//32]	(1, 8)
Dropout	[*B*, 32, 1, *T*//32]	0.5
Global AvgPool	[*B*, 32]	
Global MaxPool	[*B*, 32]	
Concatenate	[*B*, 64]	
FC → ReLU	[*B*, 32]	
FC → ReLU	[*B*, 16]	
FC → Sigmoid	[*B*, 32]	
Channel-wise Multiply	[*B*, 32, 1, *T*//32]	
Flatten	[*B*, 32 × *T*//32]	
FC → BN → ReLU → Dropout	[*B*, 128]	*p* = 0.5
FC	[*B, K*]	

*D* = *K*×*F*×2*m* is the number of virtual channels.

#### 2.2.1 Multi-band decomposition and spatial filtering

Motor imagery induces ERD/ERS patterns primarily in the mu (8–12 Hz) and beta (12–30 Hz) bands, but the discriminative frequency bands vary across subjects (Ang et al., 2008). To avoid manual band selection, the raw EEG signals are decomposed into *F* = 6 sub-bands using a bank of bandpass filters with 4 Hz bandwidth, covering 8–12 Hz, 12–16 Hz, …, 28–32 Hz. For each trial ${\text{X}}_{n}\in {\mathbb{R}}^{C\times T}$, the filtered signal in sub-band *f* is denoted ${\text{X}}_{n}^{\left(f\right)}\in {\mathbb{R}}^{C\times T}$, where *C* is the number of channels and *T* is the number of time samples. This filter bank configuration, with 4Hz bandwidth covering 8-32Hz, is consistent with prior work (Ang et al., 2008; Mane et al., 2021) and targets the mu and beta bands where ERD/ERS is most pronounced (Pfurtscheller and Lopes da Silva, 1999).

Within each sub-band, the Common Spatial Pattern (CSP) algorithm is applied to extract discriminative spatial features. Given zero-mean signals and a binary partition into class *i* and class *j*, CSP seeks a spatial filter **w**∈ℝ^*C*×1^ that maximizes the variance ratio:

$$\begin{array}{l} J\left(\text{w}\right)=\frac{{\text{w}}^{T}\Sigma_{i}\text{w}}{{\text{w}}^{T}\Sigma_{j}\text{w}} \end{array}$$ (1)

where $\Sigma_{i}=\frac{1}{N_{i}}\overset{N_{i}}{\underset{n=1}{\sum }}{\text{X}}_{n}^{\left(f\right)}{\text{X}}_{n}^{\left(f\right)T}$ is the average spatial covariance matrix computed from the *N*_*i*_ trials of class *i* in sub-band *f*. This is solved as a generalized eigenvalue problem:

$$\begin{array}{l} \Sigma_{i}\text{w}=\lambda \Sigma_{j}\text{w} \end{array}$$ (2)

A large λ means the filter produces high variance under class *i* and low variance under class *j*; a small λ gives the opposite. The eigenvectors corresponding to the *m* largest and *m* smallest eigenvalues are concatenated into the projection matrix **W**∈ℝ^*C*×2*m*^.

Since CSP is defined for two classes, we extend it to the *K*-class setting by training *K* separate binary problems. For each class *k* (*k* = 1, …, *K*), the covariance matrix of class *k* and the pooled covariance matrix of all other classes are computed, and the generalized eigenvalue problem is solved in every sub-band. This yields a projection matrix ${\text{W}}_{k}^{\left(f\right)}\in {\mathbb{R}}^{C\times 2m}$ for each class sub-band pair.

#### 2.2.2 Multi-class spatial filter fusion

In Section 2.2.1, a separate projection matrix ${\text{W}}_{k}^{\left(f\right)}$ is obtained for each class–sub-band pair. We apply all *K* projections to every trial and concatenate the results into a single representation. For class *k* in sub-band *f*, applying ${\text{W}}_{k}^{\left(f\right)}$ to trial ${\text{X}}_{n}^{\left(f\right)}$ gives:

$$\begin{array}{l} {\text{Z}}_{n,k}^{\left(f\right)}={\text{W}}_{k}^{\left(f\right)⊤}{\text{X}}_{n}^{\left(f\right)}\in {\mathbb{R}}^{2m\times T} \end{array}$$ (3)

Rather than computing log-variance as in FBCSP, we retain the full time series so that the subsequent network can learn how signal energy evolves over time. The filtered signals from all classes and sub-bands are concatenated along the channel dimension:

$$\begin{array}{l} {\text{V}}_{n}=\text{Concat}\left({\text{Z}}_{n,1}^{\left(1\right)},\ldots ,{\text{Z}}_{n,1}^{\left(F\right)},{\text{Z}}_{n,2}^{\left(1\right)},\ldots ,{\text{Z}}_{n,K}^{\left(F\right)}\right)\in {\mathbb{R}}^{D\times T} \end{array}$$ (4)

where *D* = *K*×*F*×2*m* is the total number of virtual channels. The channels are arranged in a class–sub-band–component order: the first 2*m* channels correspond to class 1 in sub-band 1, the next 2*m* to class 1 in sub-band 2, and so on. This layout places channels sharing the same class and frequency band next to each other, which helps the subsequent depthwise convolution capture local patterns within each group. Before being fed into the network, the virtual channel representation is passed through a batch normalization layer to standardize the input distribution, mitigating amplitude differences across subjects and frequency bands.

By construction of CSP, when a trial from class *k* is projected through the class-*k* spatial filters, the top *m* components tend to produce large variance while the bottom *m* components tend to produce small variance; this pattern differs when the same trial is projected through the filters of other classes. The resulting variance profile across all virtual channels provides the basis for multi-class discrimination in the shared feature space of **V**_*n*_.

#### 2.2.3 Spatial-temporal feature extraction

**Figure 2:**
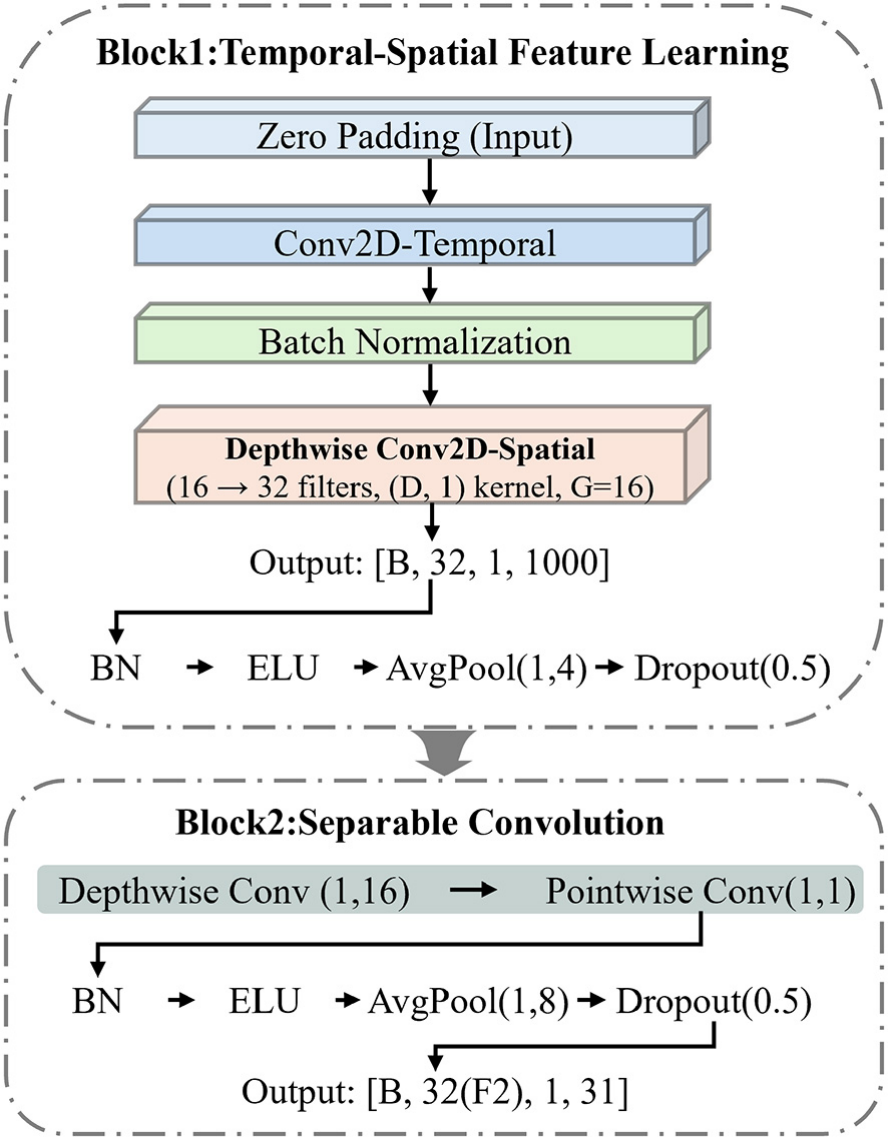
Detailed architecture of Block 1 and Block 2 in the feature extraction network.

The fused representation ${\text{V}}_{n}\in {\mathbb{R}}^{D\times T}$ is passed to a feature extraction network built on the EEGNet architecture (Lawhern et al., 2018). The backbone comprises two convolutional blocks; the detailed structure is shown in Figure 2.

Block 1 first applies a temporal convolution with kernel length of 64 samples along the time dimension, producing *F*_1_ = 16 temporal feature maps. After batch normalization, a depthwise convolution operates along the channel dimension, where each temporal feature map independently learns the combinations among the *D* virtual channels, doubling the number of feature maps to 2*F*_1_ = 32. This is followed by batch normalization, ELU activation, average pooling (stride 4), and dropout.

Block 2 applies a separable convolution to further extract temporal features: a depthwise convolution first filters along the time dimension, and a pointwise convolution then adjusts the number of feature maps to *F*_2_ = 32. This is followed by batch normalization, ELU activation, average pooling (stride 8), and dropout. Note that the depthwise convolution in the original EEGNet learns spatial filters across physical electrodes, whereas here it operates over the *D* virtual channels of **V**_*n*_. Because adjacent channels share the same class and sub-band (Section 2.2.2), the depthwise convolution captures energy contrasts within each class–sub-band group.

#### 2.2.4 Channel attention and classification

**Figure 3:**
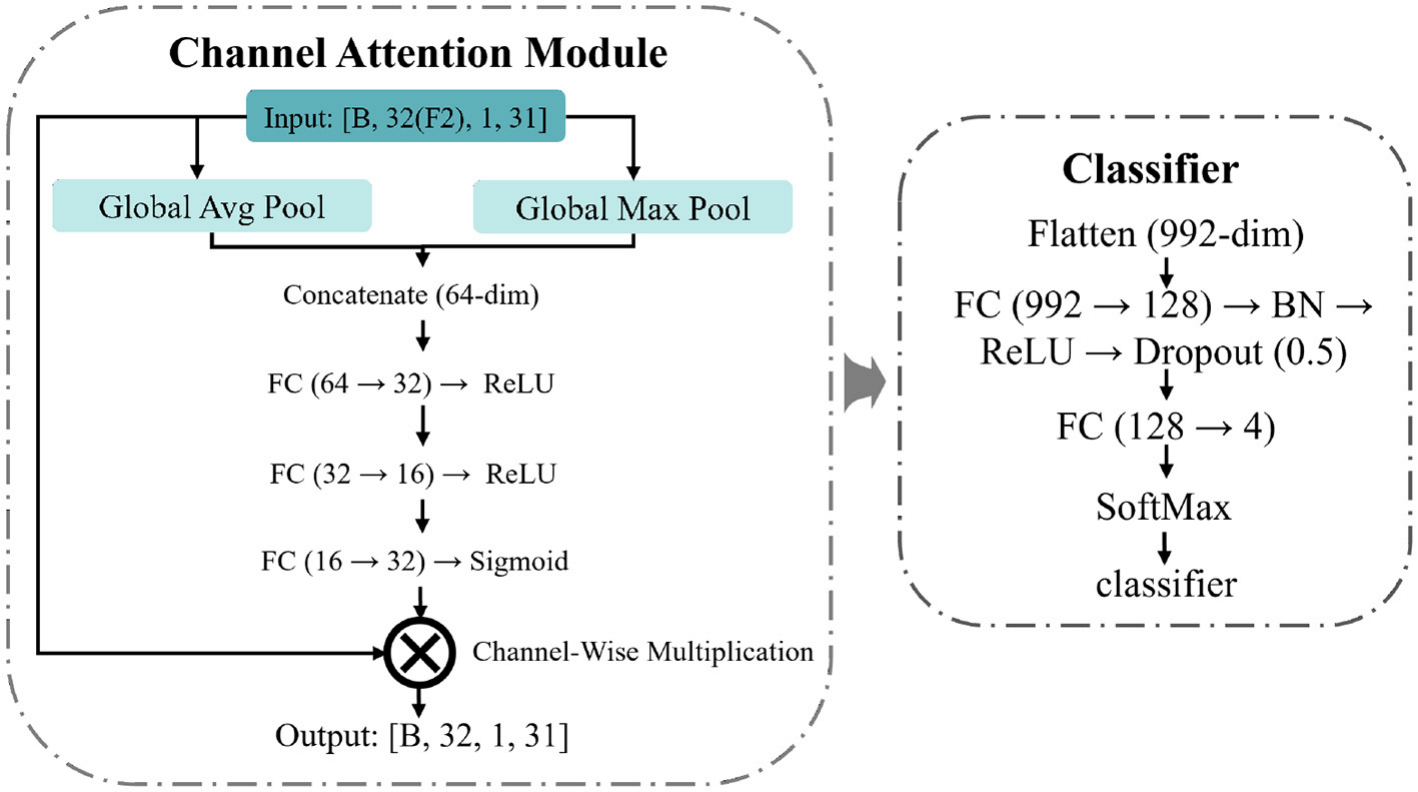
Detailed architecture of the channel attention module and classifier.

Block 2 produces *F*_2_ feature maps, but the input virtual channel contains CSP projections from all *K* classes. For a trial belonging to class *k*, the projections through class-*k* filters are expected to produce strong responses, while projections through other classes' filters may carry less relevant or redundant information. Consequently, not all feature maps contribute equally to classification. To address this, we add a channel attention module after Block 2, inspired by the channel attention mechanism in CBAM (Woo et al., 2018), to adaptively reweight the feature maps based on their relevance to the classification task. Its structure is shown in Figure 3.

The module compresses the spatial information of each feature map via global average pooling and global max pooling, and concatenates the two sets of statistics. The concatenated vector is passed through a fully connected network that first fuses the two pooling statistics back to the channel dimension, then learns inter-channel dependencies through a bottleneck structure, and finally outputs gate values via a Sigmoid function to rescale the feature maps channel-wise.

The attention-weighted feature maps are flattened and fed into a classifier consisting of fully connected layers, batch normalization, and dropout, which maps the features to *K* classes. The entire network is trained end-to-end by minimizing the cross-entropy loss:

$$\begin{array}{l} L=-\frac{1}{N}\overset{N}{\underset{n=1}{\sum }}\overset{K}{\underset{k=1}{\sum }}y_{n,k}\log\left({ŷ}_{n,k}\right), \end{array}$$ (5)

where *y*_*n, k*_ is the ground-truth label and ŷ_*n, k*_ is the predicted probability for class *k* of the *n*-th trial.

## 3 Result analysis

### 3.1 Parameter setting

The classification task in this study is a multi-class problem involving all motor imagery categories in each dataset. Five-fold cross-validation is used to split the data into training and test sets, with the mean and standard deviation of classification accuracy serving as evaluation metrics. All data-dependent operations, including band-pass filtering, CSP spatial filter computation, and network training, are performed exclusively on the training data within each fold to avoid data leakage. CSP filters are computed on the training data of each fold using a one-vs.-rest strategy for each motor imagery class, and the resulting spatial filters are applied to both the training and test sets of the corresponding fold. In addition, paired-samples t-tests are used to compare different methods, and Cohen's d is calculated to measure the effect size.

EEGNet, TTSNet, FBEEGNet, and MCFANet are trained with the same settings: learning rate of 0.001, batch size of 32, Adam optimizer, and cross-entropy loss. All hyperparameters are kept fixed across subjects and folds to avoid optimistic bias.

For individual subjects, the reported ± denotes the standard deviation across the five cross-validation folds. For the average across subjects, the ± denotes the standard deviation across subjects.

### 3.2 Compared method

In addition to CSP (Ramoser et al., 2000), FBCSP (Ang et al., 2008), and EEGNet (Lawhern et al., 2018), which are well-established baselines, we include the following methods for comparison:

**STRCA** The standard task-related component analysis method (Jia et al., 2023) applies TRCA spatial filters to remove task-unrelated components from EEG signals, and classifies trials by computing correlation coefficients between the filtered signals and class-specific templates.

**TTSNet** The two-stage-training temporal–spectral network (Jia et al., 2024) builds upon STRCA by replacing the correlation-based temporal decoding with EEGNet, and concatenates features from multiple filter banks for classification.

**FBEEGNet** This method uses the same filter bank decomposition and CSP spatial filtering as MCFANet, but applies only a single set of CSP filters to project all trials, without combining CSP outputs from multiple classes. The filtered time series are fed into EEGNet for classification. It serves as an ablation baseline to evaluate the contribution of the multi-class fusion strategy.

### 3.3 Hyperparameter tuning

The number of spatial filter pairs *m* is a key hyperparameter that needs to be determined for each dataset which is selected via grid search over *m*∈{1, 2, 3, 4, 5}. For each candidate value of *m*, the full pipeline is executed, and the value yielding the highest mean cross-validation accuracy is selected. This procedure is performed independently for each dataset to account for differences in channel count and sample size. The search range for *m* is bounded by the spatial dimensionality of the data: since CSP selects the top *m* and bottom *m* eigenvectors from *C*×*C* covariance matrices, *m* is upper-bounded by *C*/2, but eigenvalues decay rapidly from both extremes and intermediate components carry little discriminative information. In prior work, (Ang et al. 2008) used *m* = 2 for a 22-channel dataset and *m* = 1 for a 3-channel dataset, confirming that small values of *m* are generally sufficient. We therefore search *m*∈{1, 2, 3, 4, 5}, which covers the empirically effective range reported in the literature.

**Figure 4:**
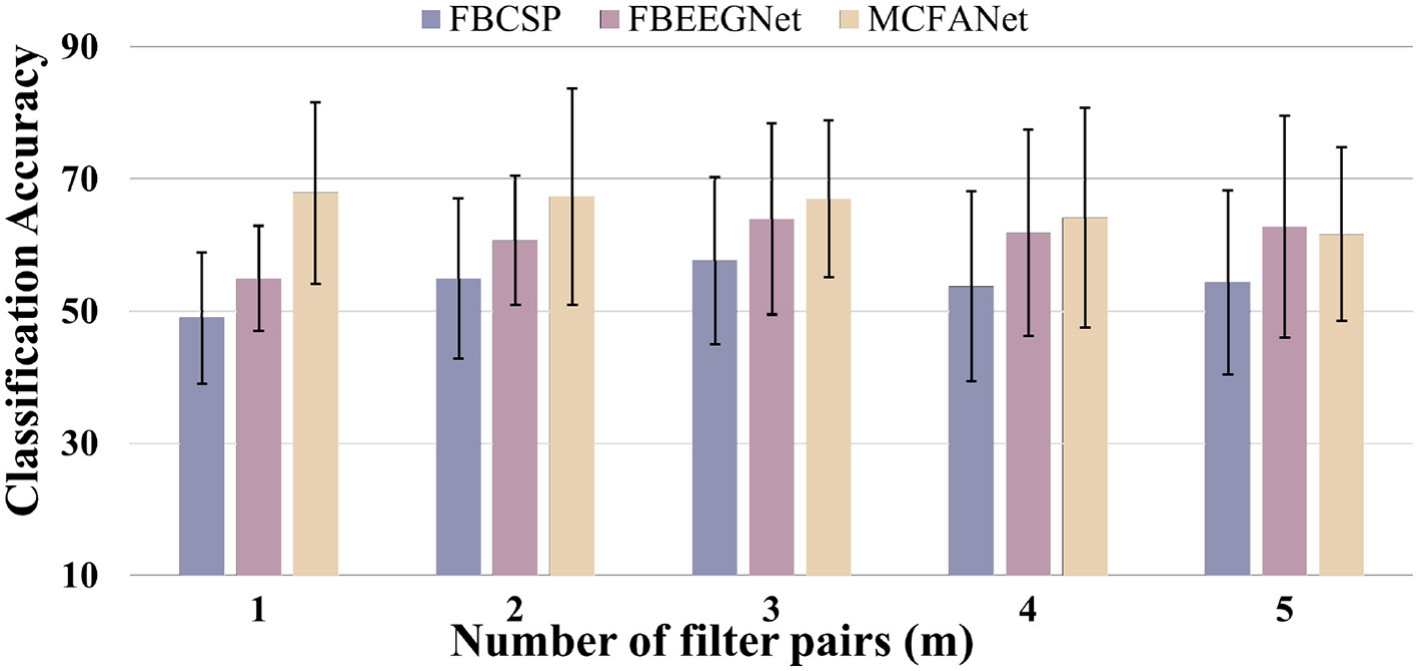
Average Classification accuracy of FBCSP, FBEEGNet, and MCFANet on BCI Competition IV Dataset 2a as a function of the number of spatial filter pairs (*m*). Error bars indicate the standard deviation across subjects.

Increasing *m* has two competing effects: additional filter pairs can capture complementary spatial patterns, but lower-ranked eigenvectors carry weaker discriminative information and are more susceptible to estimation noise, particularly when training samples are limited relative to the number of channels. On Dataset 2a, as shown in Figure 4, which has only 22 channels and 72 trials per class, covariance estimates are less reliable and the spatial dimensionality is limited, so noise from redundant components quickly offsets any benefit. MCFANet achieves its best accuracy of 67.94% at *m* = 1 and declines steadily to 61.69% at *m* = 5. FBCSP and FBEEGNet both peak at *m* = 3 (57.70% and 63.98%, respectively) before declining.

**Figure 5:**
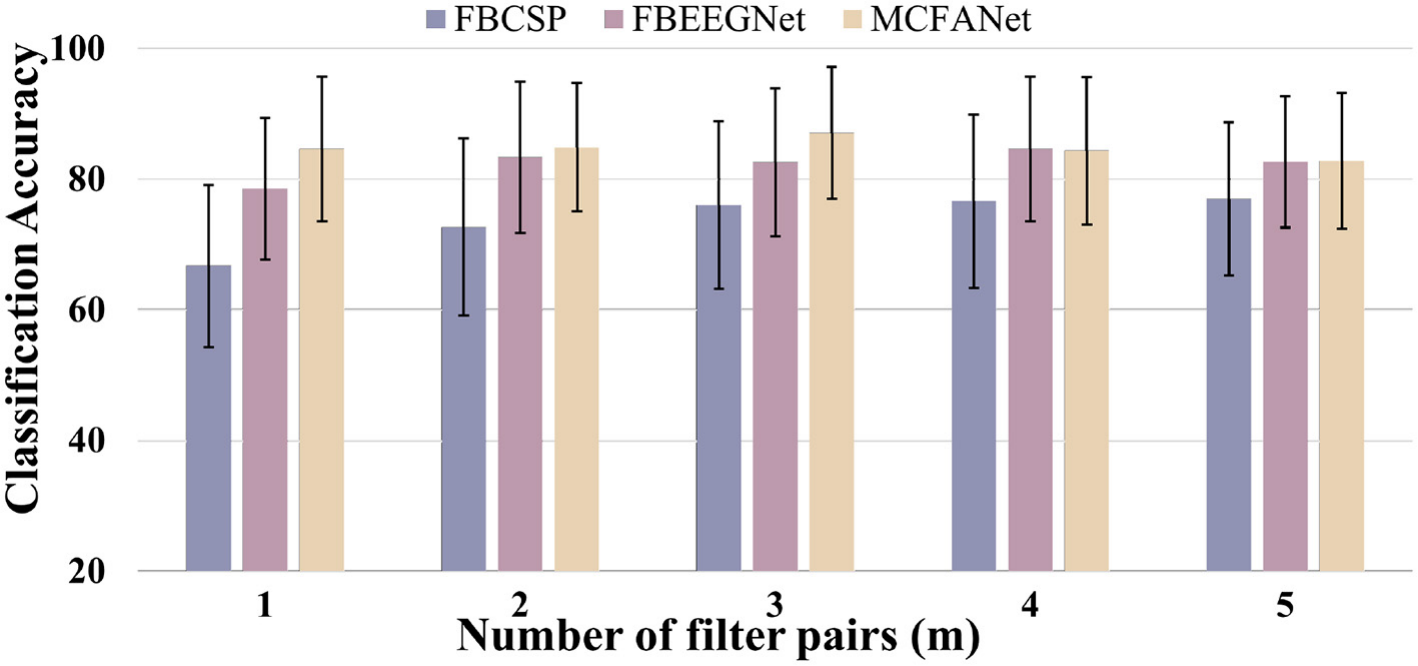
Average classification accuracy of FBCSP, FBEEGNet, and MCFANet on High Gamma Dataset as a function of the number of spatial filter pairs (*m*). Error bars indicate the standard deviation across subjects.

On HGD (Figure 5), which has 44 channels and approximately 220 trials per class, more reliable covariance estimation and richer spatial information allow additional filter pairs to contribute useful patterns before noise begins to dominate. FBCSP accuracy rises steadily from 66.75% (*m* = 1) to 77.01% (*m* = 5), FBEEGNet peaks at 84.64% with *m* = 4, and MCFANet peaks at 87.10% with *m* = 3 before declining to 82.80% at *m* = 5. These results indicate that the optimal *m* depends on the interplay between spatial dimensionality and sample size, and should be determined for each dataset using the cross-validation procedure described above. It is also worth noting that for a given *m*, MCFANet produces *K* times more virtual channels than FBEEGNet due to multi-class fusion, which explains why MCFANet reaches its optimal accuracy at a smaller *m* than the other two methods on Dataset 2a.

#### 3.3.1 Dimension matched comparison

To verify that the performance gain of MCFANet is not simply a result of increased input dimensionality, we matched the virtual channel size *D* across both methods by adjusting *m* accordingly. As shown in Table 2, MCFANet outperforms FBEEGNet at all three dimensionality levels on Dataset 2a, and at *D* = 96 and *D* = 144 on HGD. The entry for FBEEGNet at *D* = 144 on Dataset 2a is unavailable because the required CSP components (2*m* = 24) exceed the number of EEG channels (*C* = 22).

**Table 2:** Dimension matched accuracy comparison between FBEEGNet and MCFANet.

*D*	FBEEGNet		MCFANet	
	*m*	Accuracy (%)	*m*	Accuracy (%)
BCI competition IV dataset 2a.				
48	4	61.88 ± 15.60	1	**67.94 ± 13.70**
96	8	62.99 ± 16.01	2	**67.39 ± 16.34**
144	12	—	3	**67.05 ± 11.87**
High gamma dataset				
48	4	84.64 ± 11.03	1	84.61 ± 11.06
96	8	84.06 ± 11.55	2	**84.91 ± 9.86**
144	12	83.96 ± 11.52	3	**87.10 ± 10.09**

Accuracy is reported as mean ± standard deviation (%) across subjects. Bold values indicate the best performance.

### 3.4 Classification performance

In the following comparisons, each method uses the value of *m* that yields its highest cross-validation accuracy on the respective dataset, as determined by the grid search procedure in Section 3.3. Specifically, on Dataset 2a, MCFANet uses *m* = 1, FBEEGNet and FBCSP use *m* = 3; on HGD, MCFANet uses *m* = 3, FBEEGNet uses *m* = 4, and FBCSP uses *m* = 5. The same settings apply to all subsequent results.

**Figure 6:**
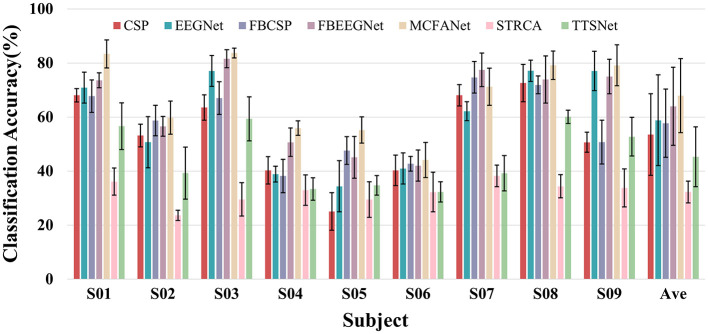
Four-class classification performance comparison between MCFANet and other methods on BCI Competition IV Dataset 2a. The rightmost group (Ave) denotes the mean accuracy and standard deviation across all nine subjects. Error bars indicate the standard deviation across five folds for individual subjects, and across subjects for the average (Ave).

In this section, six methods are compared to the proposed MCFANet method, including CSP, FBCSP, STRCA, TTSNet, EEGNet, and FBEEGNet. Figure 6 shows the classification accuracy of seven methods for each subject on the BCI Competition IV Dataset 2a, along with the mean accuracy and standard deviation.

On this dataset, MCFANet achieves a mean accuracy of 67.94% with a standard deviation of 13.70, outperforming FBEEGNet (63.98% ± 14.43) and EEGNet (58.79% ± 16.78). Traditional machine learning methods all perform worse than MCFANet, with STRCA showing the lowest accuracy of only 32.25% ± 4.04. Although TTSNet is a deep learning method, it performs poorly on motor imagery classification (45.28% ± 11.04), only better than STRCA.

According to the performance variation across subjects, MCFANet shows good generalization ability and stability. Among the 9 subjects, MCFANet achieves the highest accuracy in 8 subjects (S01, S02, S03, S04, S05, S06, S08, S09). For the three weakest subjects (S04–S06), MCFANet still improves over the second-best method by 2–10 percentage points.

Table 3 summarizes the classification performance of all methods on the HGD dataset with 14 subjects. MCFANet ranks first with a mean accuracy of 87.10% ± 10.09, improving 2.46 percentage points over FBEEGNet (84.64% ± 11.03). Deep learning methods perform better than traditional methods overall, with EEGNet performing well (83.31%), while STRCA nearly fails on this dataset (33.41%). At the subject level, MCFANet achieves the best performance in 13 out of 14 subjects. For subjects where all methods perform well (S03, S04, S12, S14), MCFANet further improves the accuracy to 96%-98%, with S14 reaching the highest at 98.64%. For subjects with poor performance (e.g., S09, S11), MCFANet still holds a relative advantage: on S09, it reaches 61.93%, ahead of EEGNet (60.91%) and FBEEGNet (58.18%); on S11, it achieves 72.73%, slightly better than EEGNet (71.93%). FBEEGNet only outperforms MCFANet on S13 (86.50% vs 85.12%), indicating that MCFANet maintains a stable advantage across most subjects.

**Table 3:** Comparison of 4-class classification accuracy (%) on high gamma dataset across 14 subjects.

Subject	CSP	FBCSP	STRCA	EEGNet	TTSNet	FBEEGNet	MCFANet
S01	66.46 ± 4.66	72.73 ± 3.17	26.96 ± 2.35	69.29 ± 2.78	66.45 ± 3.31	76.19 ± 5.12	**80.26 ± 6.19**
S02	61.03 ± 4.02	71.40 ± 2.61	39.09 ± 2.55	86.68 ± 2.62	75.95 ± 2.13	82.11 ± 3.17	**88.04 ± 4.38**
S03	74.09 ± 7.87	89.89 ± 2.01	33.86 ± 2.89	95.57 ± 0.98	93.52 ± 1.37	94.89 ± 1.87	**97.39 ± 0.92**
S04	76.65 ± 4.94	89.50 ± 0.96	34.86 ± 3.61	95.75 ± 1.15	92.96 ± 1.60	96.09 ± 1.00	**96.98 ± 1.35**
S05	59.24 ± 3.86	78.72 ± 3.04	31.58 ± 3.39	83.45 ± 3.54	73.71 ± 2.37	89.01 ± 2.92	**90.54 ± 0.71**
S06	58.72 ± 5.28	75.34 ± 1.18	29.25 ± 2.74	78.21 ± 4.17	73.63 ± 2.39	75.23 ± 2.52	**80.40 ± 4.60**
S07	60.48 ± 2.14	65.63 ± 2.29	31.50 ± 1.92	80.48 ± 1.76	71.86 ± 2.76	85.39 ± 3.31	**86.83 ± 2.45**
S08	75.99 ± 3.65	85.48 ± 2.19	37.31 ± 1.23	82.58 ± 4.77	73.09 ± 2.41	87.46 ± 3.43	**91.44 ± 1.47**
S09	46.59 ± 6.65	50.00 ± 4.71	29.77 ± 2.23	60.91 ± 4.29	49.09 ± 1.71	58.18 ± 5.87	**61.93 ± 3.39**
S10	67.41 ± 2.57	82.66 ± 3.44	39.11 ± 3.86	91.15 ± 2.24	87.08 ± 2.11	92.00 ± 2.14	**92.25 ± 1.25**
S11	57.95 ± 2.85	62.16 ± 2.84	36.93 ± 2.57	71.93 ± 3.76	67.27 ± 2.35	69.20 ± 3.78	**72.73 ± 1.65**
S12	78.90 ± 3.09	90.19 ± 2.46	38.99 ± 3.93	93.61 ± 3.08	93.62 ± 2.74	95.33 ± 1.15	**96.81 ± 1.17**
S13	53.87 ± 8.19	74.13 ± 2.81	32.62 ± 1.95	81.25 ± 3.04	77.62 ± 2.32	**86.50 ± 2.81**	85.12 ± 2.07
S14	71.82 ± 6.39	90.34 ± 1.57	25.91 ± 3.88	95.45 ± 2.16	95.00 ± 0.84	97.39 ± 1.05	**98.64 ± 0.45**
**Ave**	64.94 ± 9.29	77.01 ± 11.71	33.41 ± 4.32	83.31 ± 10.34	77.92 ± 12.73	84.64 ± 11.03	**87.10 ± 10.09**

For each subject, ± indicates the standard deviation across five folds. For the average row (Ave), ± indicates the standard deviation across subjects. Bold values indicate the best performance.

Compared with the BCI Competition IV 2a dataset, all methods achieve higher overall accuracy on HGD with smaller standard deviations, suggesting less inter-subject variability in this dataset. TTSNet shows clear improvement on HGD relative to the 2a dataset, while STRCA performs poorly on both datasets, indicating its difficulty in handling motor imagery classification tasks. Despite the different characteristics of the two datasets, MCFANet achieves the best performance on both.

#### 3.4.1 Statistical analysis

To further test whether the performance improvement of MCFANet over baseline methods is statistically meaningful, we used paired-samples t-tests to analyze the differences in accuracy between methods, and calculated Cohen's d to measure the actual size of the differences.

As shown in Table 4a, MCFANet differs significantly from all compared methods (*p* < 0.05), with all effect sizes reaching the large level (|*d*|≥0.8). On Dataset 2a, the comparison with FBEEGNet yields the smallest effect size (*d* = 0.82), while on HGD, all comparisons reach *p* < 0.001 with the larger sample size providing greater statistical power. Table 4b presents the paired-samples t-test and Cohen's d effect size results on the HGD dataset. MCFANet differs significantly from all compared methods (*p* < 0.05), with all effect sizes reaching the large effect level (|*d*|≥0.8). Specifically, compared to traditional spatial filtering methods CSP and FBCSP, the differences are highly significant (*p* < 0.001), with effect sizes of 3.67 and 2.29 respectively, showing that MCFANet has clear advantages in feature extraction. Compared to STRCA, MCFANet also shows a highly significant difference (*p* < 0.001, *d* = 5.19), which further confirms the limitations of STRCA on motor imagery classification.

**Table 4:** Paired *t*-test *p*-values and Cohen's *d* effect sizes of MCFANet vs. other methods.

Comparison	*p*-value	Cohen's *d*
A BCI competition IV dataset 2a.		
vs. CSP	0.0029^**^	1.4069
vs. FBCSP	0.0163^*^	1.0097
vs. EEGNet	0.0032^**^	1.3808
vs. TTSNet	< 0.001^***^	3.9773
vs. FBEEGNet	0.0393^*^	0.8199
vs. STRCA	< 0.001^***^	2.6168
B High gamma dataset		
vs. CSP	< 0.001^***^	3.6652
vs. FBCSP	< 0.001^***^	2.2920
vs. EEGNet	< 0.001^***^	1.1623
vs. TTSNet	< 0.001^***^	1.6992
vs. FBEEGNet	< 0.001^***^	1.2106
vs. STRCA	< 0.001^***^	5.1873

^*^p < 0.05,

^**^p < 0.01,

^***^p < 0.001; effect size: |*d*| < 0.5 (small), 0.5 ≤ |*d*| < 0.8 (medium), |*d*|≥0.8 (large).

Compared to deep learning methods EEGNet, TTSNet, and FBEEGNet, MCFANet reaches statistical significance in all cases. The comparison with EEGNet yields *p* < 0.001 with an effect size of 1.16, while comparisons with TTSNet and FBEEGNet both reach *p* < 0.001, with effect sizes of 1.70 and 1.21 respectively. With the larger sample size (n=14 vs n=9) and more channels, the p-values are generally lower and the effect sizes are larger overall compared to the BCI Competition IV 2a dataset.

#### 3.4.2 F1 score analysis

Table 5 shows the per-class F1 scores and macro-F1 score of all compared methods on Dataset 2a. MCFANet achieves the highest macro-F1 of 67.72% ± 13.84, outperforming FBEEGNet (63.32% ± 14.63) and EEGNet (58.54% ± 16.82), consistent with the accuracy results in Figure 6. At the class level, MCFANet obtains the best F1 on Right Hand (66.18%), Foot (68.18%), and Tongue (68.23%), while FBEEGNet achieves a marginally higher F1 on Left Hand (70.20% vs. 68.31%).

**Table 5:** Per-class F1 scores and macro-F1 score (%) of all compared methods on BCI Competition IV Dataset 2a.

Class	CSP	FBCSP	EEGNet	FBEEGNet	MCFANet	STRCA	TTSNet
Left	58.04	57.25	59.51	**70.20**	68.31	27.58	49.70
Right	52.53	57.28	57.20	58.27	**66.18**	30.82	44.27
Foot	51.89	57.14	58.05	57.87	**68.18**	30.36	36.39
Tongue	51.62	59.07	59.40	66.94	**68.23**	39.03	46.17
**Ave**	53.52 ± 15.33	57.69 ± 12.74	58.54 ± 16.82	63.32 ± 14.63	**67.72 ± 13.84**	31.95 ± 4.13	44.13 ± 10.97

For the average row (Ave), ± indicates the standard deviation across subjects.

Bold values indicate the best performance.

Table 6 shows the per-class F1 scores and macro-F1 on the HGD dataset. MCFANet reaches a macro-F1 of 87.16% ± 9.99, ahead of FBEEGNet (84.69% ± 10.90) and EEGNet (83.29% ± 10.33). Unlike on Dataset 2a, MCFANet obtains the best F1 score across all four classes. Rest and Foot show higher F1 scores than the two hand classes across all methods; Left and Right Hand remain the hardest to distinguish, though MCFANet still leads on both (82.33% and 83.15%).

**Table 6:** Comparison of per-class and macro-F1 scores (%) on High Gamma Dataset.

Class	CSP	FBCSP	EEGNet	FBEEGNet	MCFANet	STRCA	TTSNet
Left	62.45	70.60	76.70	81.65	**82.33**	31.59	71.10
Right	64.03	75.66	78.34	80.07	**83.15**	32.04	71.95
Foot	68.38	81.15	88.78	88.95	**91.54**	38.05	84.01
Rest	65.05	80.12	89.36	88.08	**91.61**	32.12	84.52
**Ave**	64.98 ± 9.28	76.88 ± 11.77	83.29 ± 10.33	84.69 ± 10.90	**87.16 ± 9.99**	33.45 ± 4.37	77.90 ± 12.70

For the average row (Ave), ± indicates the standard deviation across subjects. Bold values indicate the best performance.

#### 3.4.3 Computational cost

To evaluate the feasibility of real-time deployment, we measured the single-trial inference time of each deep learning method on CPU (Intel i7-12700H). Each model was run 1,000 times and the average time was recorded. As shown in Table 7, on Dataset 2a, MCFANet requires 1.87 ms per trial, compared to 1.10 ms for EEGNet and 1.21 ms for FBEEGNet. On HGD, the inference time of MCFANet increases to 3.12 ms due to the larger number of virtual channels (144 vs 48), but remains well below the typical BCI feedback window of several hundred milliseconds. TTSNet is the slowest method on both datasets (6.92 ms and 8.26 ms), as it runs a separate EEGNet branch for each frequency band.

**Table 7:** Single-trial inference time (ms) of deep learning methods.

Dataset	Model	Time (ms)	Dataset	Model	Time (ms)
Dataset 2a	EEGNet	1.101	HGD	EEGNet	1.247
	FBEEGNet (*m* = 3)	1.211		FBEEGNet (*m* = 4)	1.270
	TTSNet	6.916		TTSNet	8.261
	MCFANet (*m* = 1)	1.866		MCFANet (*m* = 3)	3.116

### 3.5 Confusion matrix analysis

To evaluate the classification performance of MCFANet, we plot the aggregated confusion matrix across all subjects. Confusion matrices are computed per subject by concatenating predictions across all five folds, and then summed across subjects. Each element in the matrix shows both the sample count and the row-normalized percentage, where each element represents the percentage of samples from a given true class that are classified into each predicted class.

**Figure 7:**
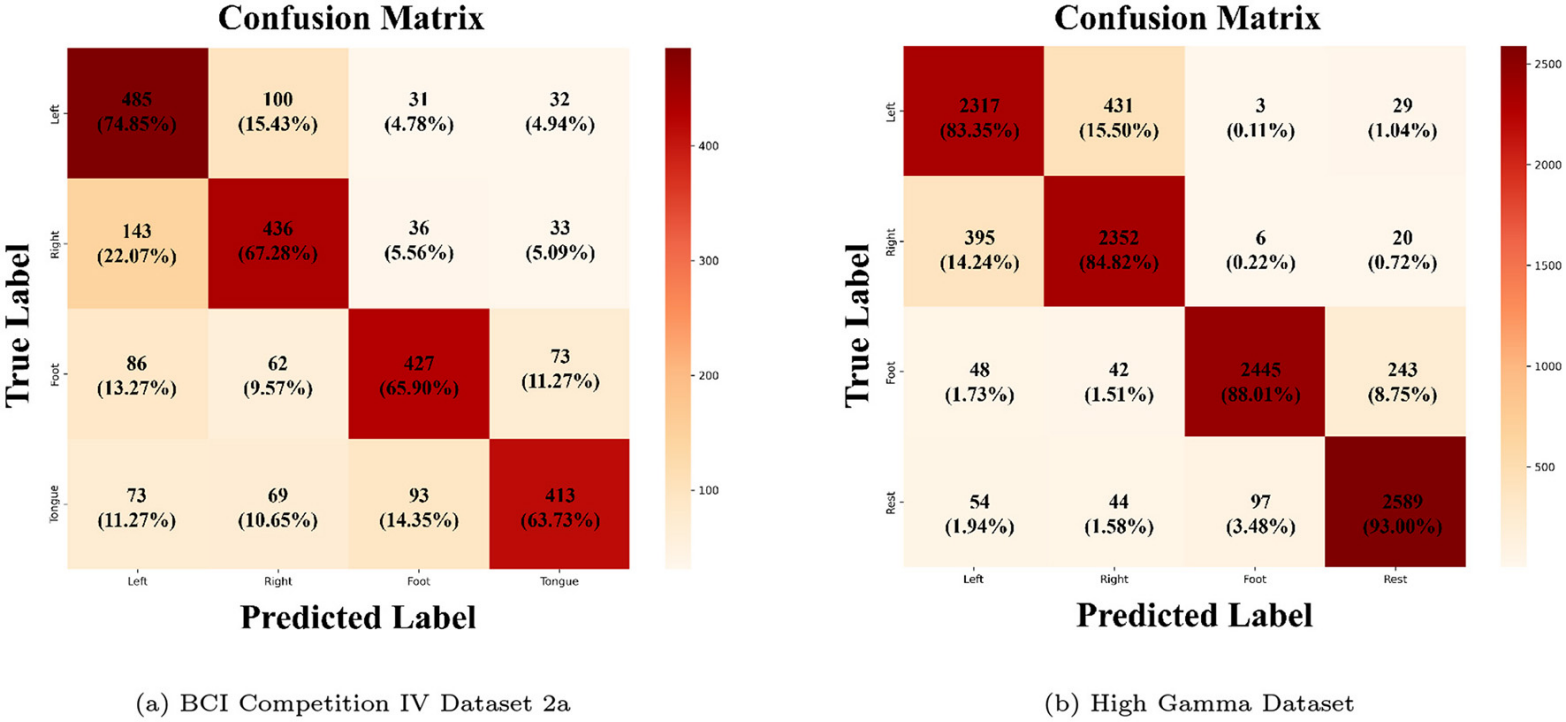
Aggregated confusion matrices of MCFANet on the 2a dataset (left) and the HGD dataset (right). **(a)** BCI Competition IV Dataset 2a. **(b)** High Gamma Dataset.

Figure 7a shows the aggregated confusion matrix of MCFANet across 9 subjects on the BCI Competition IV 2a dataset. Among the four classes, Left Hand has the highest accuracy (74.85%), followed by Right Hand (67.28%), while Foot and Tongue are lower at 65.90% and 63.73%. The main confusion occurs between Left Hand and Right Hand: 22.07% of Right Hand trials are misclassified as Left Hand, and 15.43% of Left Hand trials are misclassified as Right Hand. This is expected given the similarity of left and right hand motor imagery in EEG signals. Foot and Tongue also show some mutual confusion (14.35% and 11.27%), likely because both involve midline EEG activity. By comparison, confusion across different body parts (e.g., Left Hand vs Foot, Right Hand vs Tongue) is much smaller, showing that MCFANet can better separate motor imagery tasks that have different spatial patterns.

Figure 7b shows the aggregated confusion matrix of MCFANet across 14 subjects on the HGD dataset. Unlike the 2a dataset, HGD includes Rest instead of Tongue, which changes the confusion pattern. Rest, as a non-motor state, differs clearly from the three motor tasks and reaches the highest accuracy (93.00%), with misclassification from other classes all below 4%. Foot reaches 88.01%, with most of its errors going to Rest (8.75%), matching the overlap seen in the t-SNE visualization. Left Hand and Right Hand reach 83.35% and 84.82%, and their mutual confusion (15.50% and 14.24%) remains the main source of errors on this dataset. However, hand tasks are rarely confused with Foot or Rest (all below 2%), showing that MCFANet can effectively separate tasks with different spatial patterns.

Across the two datasets, left–right hand confusion is the main error source on both, which reflects the inherent similarity between left and right hand motor imagery. The HGD dataset shows higher accuracy overall, with errors more focused between the two hand classes. This likely comes from the higher channel count, which gives MCFANet more spatial information to work with.

### 3.6 T-SNE feature visualization

**Figure 8:**
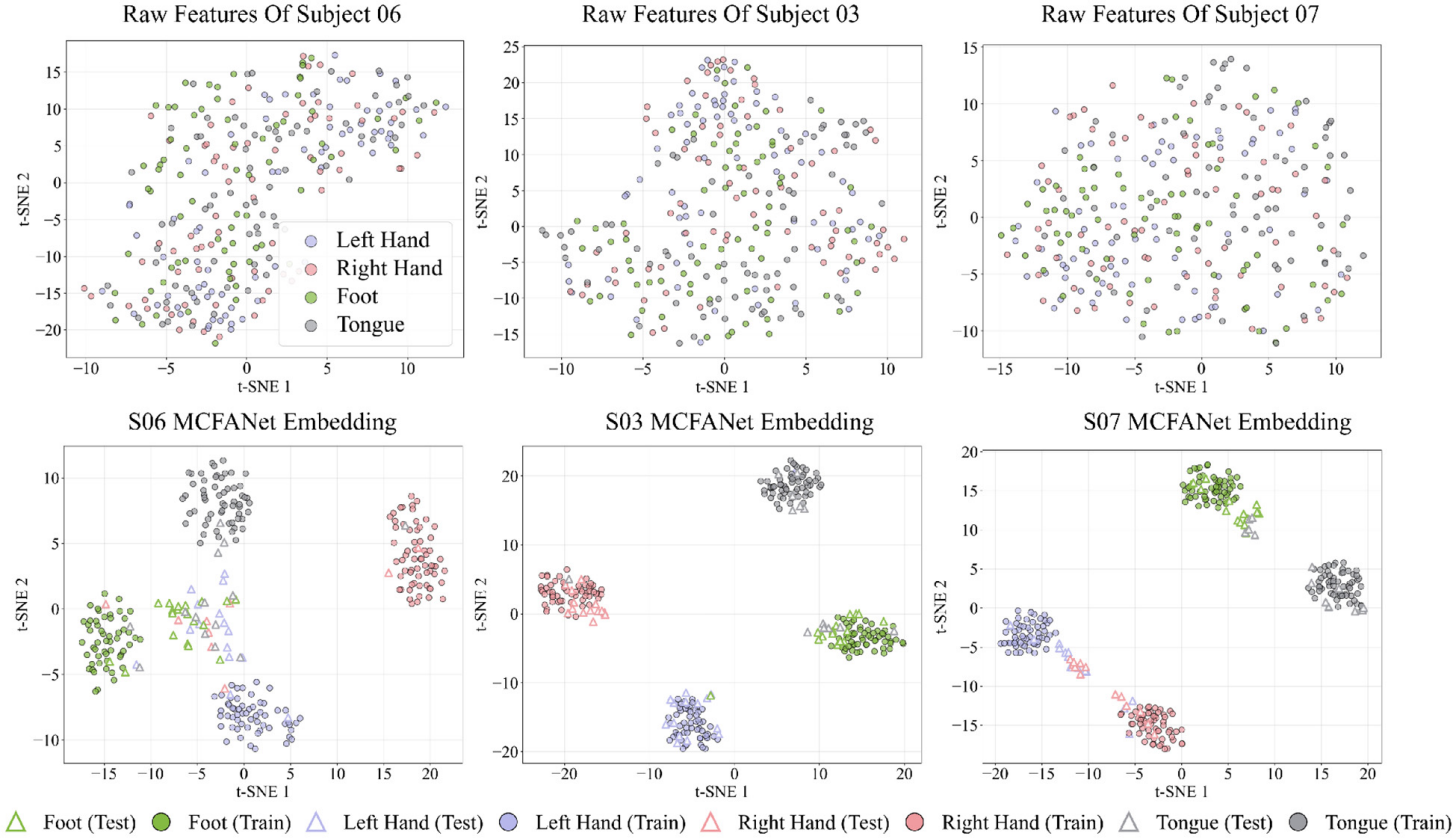
t-SNE visualizations of raw features (top row) and MCFANet learned embeddings (bottom row) for three representative subjects from BCI Competition IV Dataset 2a: S06 (lowest accuracy), S03 (highest accuracy), and S07 (closest to average).

**Figure 9:**
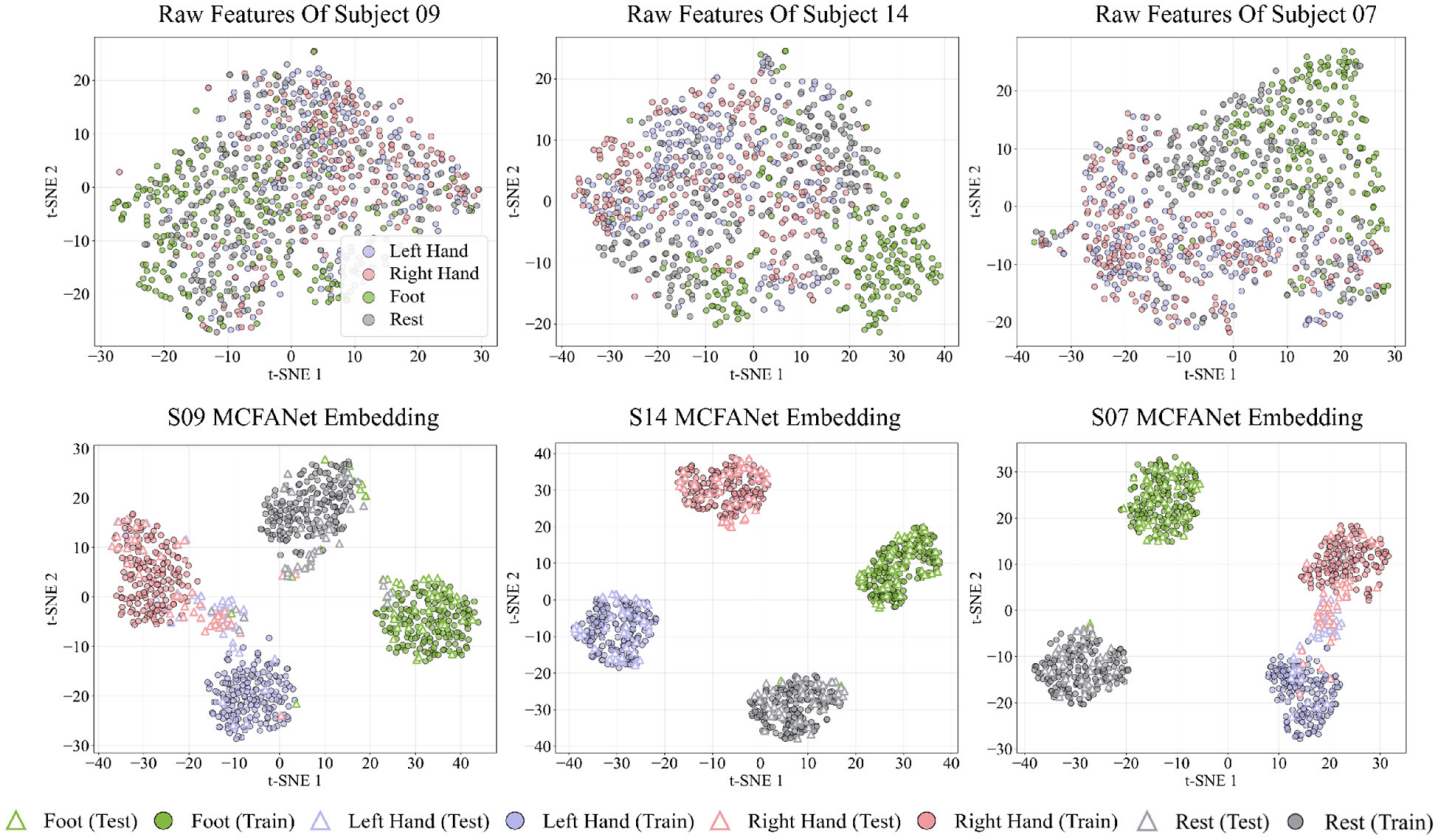
t-SNE visualizations of raw features (top row) and MCFANet learned embeddings (bottom row) for three representative subjects from High Gamma Dataset: S09 (lowest accuracy), S14 (highest accuracy), and S07 (closest to average).

Figures 8, 9 show the t-SNE visualizations of MCFANet on the BCI Competition IV-2a and HGD datasets, respectively, to evaluate the model's improvement in feature separability. For each subject, the t-SNE visualization is based on the fold whose classification accuracy is closest to the subject-level mean across all five folds. Three representative subjects are selected per dataset: the one with the highest accuracy, the lowest accuracy, and the closest to the overall mean accuracy

The raw features are extracted as follows: the 8-32 Hz frequency range is divided into 6 sub-bands (8-12 Hz, 12-16 Hz,… , 28-32 Hz). For each trial, the power spectral density of each channel is estimated using the Welch method, and the power in each sub-band is calculated through numerical integration, resulting in a *C*×6 dimensional feature vector (where C is the number of channels). The deep embeddings are obtained from the output of the layer before the classifier, with a dimension of 128. For comparison, the embeddings from both training and test sets were combined and mapped to a two-dimensional plane using t-SNE. In the figures, training samples are shown as filled circles, test samples as hollow triangles, and different classes are marked with different colors, providing a clear view of the model's ability to distinguish features in both training and test data.

Across both datasets, the raw features show heavy overlap among classes, with no clear boundaries between them. After processing by MCFANet, training samples form compact and separated clusters, indicating that the network has learned discriminative representations. The separation of test samples follows classification accuracy: for high-accuracy subjects (S03 on Dataset 2a, 84%; S14 on HGD, 98%), test points fall within the corresponding training clusters; for average-accuracy subjects (S07, 71%; S07, 87%), most classes remain separable but some overlap appears; for low-accuracy subjects (S06, 44%; S09, 62%), inter-class confusion is visible in the test distribution.

On both datasets, the main overlap occurs between Left Hand and Right Hand, which is expected given their similar cortical representations and is consistent with the confusion matrix analysis in Section 3.5. On HGD, Rest forms an isolated cluster separate from all motor classes, and Foot also forms a clearly separated cluster across subjects, benefiting from its distinct spatial pattern.

### 3.7 Gradient-based interpretability analysis

To examine whether the network learns to prioritize specific frequency bands or class-specific spatial responses, we performed a gradient-based interpretability analysis on the trained MCFANet. For each test trial, we computed the gradient of the target-class logit with respect to the input virtual channels. The absolute gradient value of each channel, averaged over the time dimension, serves as a measure of how much the network's classification decision depends on that channel. Because the virtual channels have a known structure–each channel corresponds to a specific CSP filter class and frequency sub-band–the per-channel importance scores can be aggregated by frequency sub-band (six bands from 8–12 Hz to 28–32 Hz) and by CSP filter class (four motor imagery classes) to reveal which spectral and spatial components the network relies on most. This analysis was conducted on Dataset 2a using the fold whose classification accuracy was closest to the mean accuracy across the five cross-validation folds for each subject, with importance scores normalized within each subject.

**Figure 10:**
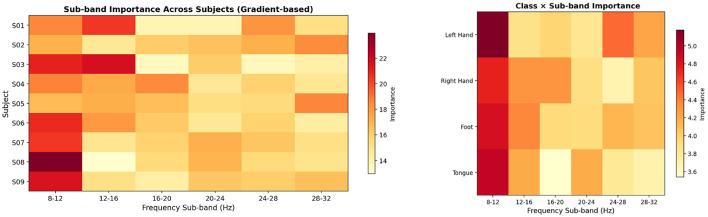
Gradient-based importance of input virtual channels on Dataset 2a. **Left**: relative importance (%) of each frequency sub-band for each subject, normalized within each subject. **Right**: importance aggregated by CSP filter class and frequency sub-band, averaged across all nine subjects. Darker colors indicate higher importance.

Figure 10 presents the gradient-based importance analysis across frequency sub-bands and CSP filter classes. The left figure shows that the 8–12 Hz sub-band has the highest importance for seven of the nine subjects, which is expected since motor imagery signals are mainly carried by mu-band activity. The right figure shows that this pattern holds across all four classes, and that the four CSP filter classes receive similar importance overall, suggesting that the network uses spatial information from all classes rather than depending on one specific class.

## 4 Discussion

A fundamental limitation of existing multi-class motor imagery (MI) decoding methods lies in the absence of a unified representation that simultaneously captures class-specific spatial discriminative responses. Traditional CSP-based approaches extend binary CSP to multi-class settings through one-versus-rest decomposition, where each subproblem is optimized independently in a separate feature space. As a result, the relative relationships among class-conditioned responses are not explicitly modeled and are typically integrated only at the decision level. The MCFANet framework addresses this limitation by constructing a shared feature space in which the responses of a single trial under all class-specific spatial filters coexist, enabling the network to jointly process the responses of all classes. This is particularly relevant in multi-class MI scenarios where cortical activations partially overlap across classes, making subtle spatial contrasts difficult to capture when classes are treated independently.

An essential design aspect of MCFANet is the retention of the full time series after CSP projection, rather than compressing spatially filtered signals into log-variance features as in standard FBCSP pipelines. Although log-variance provides a compact summary of signal energy, it removes temporal information that is known to be informative in MI tasks. Event-related desynchronization and synchronization (ERD/ERS) patterns differ across classes not only in magnitude but also in onset latency, temporal evolution, and duration. Preserving the CSP-projected time series allows subsequent convolutional layers to model these dynamics within a spatially optimized subspace, effectively decoupling spatial discrimination from temporal feature learning and shifting feature compression from a fixed statistical operation to a data-driven stage.

The multi-class fusion strategy is complemented by a channel attention mechanism that addresses the uneven contribution of feature maps introduced by concatenating class- and sub-band-specific CSP outputs. Because projections associated with non-target classes may still respond to a given trial, not all virtual channels contribute equally to classification. The attention module enables adaptive reweighting of feature maps based on their relevance, acting as a soft selection mechanism rather than enforcing hard pruning.

STRCA achieved near-chance level accuracy on both datasets, which can be attributed to its core mechanism. STRCA applies TRCA spatial filters to maximize inter-trial signal reproducibility and classifies trials by computing correlation coefficients between the filtered signals and class-specific templates. Its effectiveness therefore relies on the presence of phase-locked, temporally consistent waveforms across trials of the same class. However, the discriminative information in motor imagery resides primarily in non-phase-locked power modulations in the mu/beta bands (ERD/ERS), with no phase-locked component shared across trials. Consequently, the spatial filters derived by TRCA fail to extract discriminative features from MI signals, and the template-based correlation becomes ineffective. TTSNet partially compensates for this limitation by replacing the correlation-based temporal decoding with EEGNet, which accounts for its improved accuracy on HGD. Nevertheless, its spatial filtering stage still relies on TRCA, constraining overall performance. This contrast suggests that effective spatial filtering for MI classification should maximize inter-class variance rather than inter-trial reproducibility, which is precisely the optimization objective of CSP.

FBEEGNet and MCFANet share the same filter-bank decomposition and CSP spatial filtering; the difference is that FBEEGNet projects all trials through only a single set of CSP filters, yielding a virtual channel dimensionality of *F*×2*m*, whereas MCFANet concatenates the CSP outputs from all K classes, expanding the dimensionality to *K*×*F*×2*m*. This difference leads to improvements of approximately 4 and 2.5 percentage points on Dataset 2a and HGD, respectively. The benefit of multi-class fusion is not simply a matter of larger input dimensionality, but rather that the network can simultaneously observe how the same trial responds under different class-specific filters, which provides inter-class contrast information that a single set of filters cannot offer.

The two datasets yield different optimal values of *m*: *m* = 1 for Dataset 2a and *m* = 3 for HGD. Dataset 2a has only 22 channels, so the spatial contrasts captured by lower-ranked filter pairs are weak and prone to fitting noise in the training data, making a single pair sufficient. HGD, with 44 channels and more training trials per class, provides enough spatial dimensionality and data to support multiple filter pairs that encode complementary contrasts without overfitting.

It is also important to note that the two datasets differ in their fourth class: Dataset 2a includes tongue imagery, whereas HGD includes a resting state. Because rest involves no motor-related ERD/ERS in the sensorimotor cortex, it is easier to distinguish from the other three active classes. This is reflected in the confusion matrices, where Rest achieves the highest per-class accuracy of 93.00% with minimal confusion, while Tongue in Dataset 2a reaches only 63.73%. Therefore, direct comparison of absolute accuracy between the two datasets should be interpreted with caution.

Several limitations should be noted. The virtual channel dimensionality *D* = *K*×*F*×2*m* grows with the number of classes, sub-bands, and filter pairs, reaching 144 for *K* = 4, *F* = 6, *m* = 3, which adds considerable computational cost to the network and may constrain real time deployment. In addition, CSP spatial filters are estimated from the training set covariance matrices, so if the statistical properties of the EEG shift between training and testing, for example, due to changes in attention state, muscle artifact levels, or electrode contact conditions across sessions, the discriminative power of the filters degrades. Future work may explore more compact fusion strategies or adaptive spatial filtering mechanisms to alleviate these constraints.

Overall, reorganizing class-specific spatial responses into a shared and temporally expressive feature space provides an effective bridge between traditional CSP-based pipelines and modern deep learning approaches. This perspective highlights the importance of representation structure, rather than network depth alone, in advancing multi-class MI decoding.

## 5 Conclusion

A key challenge in multi-class motor imagery decoding lies in organizing class-specific spatial discriminative information within a unified representation. To address this issue, a multi-class fusion framework was introduced that integrates the time-series responses of class-wise CSP projections across multiple frequency bands into a shared feature space, enabling the network to jointly process the responses of all classes while preserving discriminative temporal dynamics.

Experiments on two public datasets demonstrate that this representation strategy leads to consistent improvements over conventional CSP-based pipelines and existing deep learning baselines. More broadly, the results suggest that reorganizing neurophysiologically grounded spatial features into forms better aligned with end-to-end learning provides a promising direction for multi-class EEG decoding.

## Data Availability

Publicly available datasets were analyzed in this study. This data can be found here: https://bnci-horizon-2020.eu/database/data-sets; https://github.com/robintibor/high-gamma-dataset.

## References

[B1] Altaheri H. Muhammad G. Alsulaiman M. (2022). Physics-informed attention temporal convolutional network for EEG-based motor imagery classification. IEEE Trans. Indust. Inform. 19, 2249–2258. doi: 10.1109/TII.2022.3197419

[B2] AltuwaijriG. A. MuhammadG. AltaheriH. AlsulaimanM. (2022). A multi-branch convolutional neural network with squeeze-and-excitation attention blocks for EEG-based motor imagery signals classification. Diagnostics 12:995. doi: 10.3390/diagnostics1204099535454043 PMC9032940

[B3] Ang K. K. Chin Z. Y. Zhang H. Guan C. (2008). “Filter bank common spatial pattern (FBCSP) in brain-computer interface,” in Proceedings of the IEEE International Joint Conference on Neural Networks (Hong Kong: IEEE), 2390–2397.

[B4] Arvaneh M. Guan C. Ang K. K. Quek C. (2011). Optimizing the channel selection and classification accuracy in EEG-based BCI. IEEE Trans. Biomed. Eng. 58, 1865–1873. doi: 10.1109/TBME.2011.2131142 21427014

[B5] Barachant A. Bonnet S. Congedo M. Jutten C. (2012). Multiclass brain-computer interface classification by Riemannian geometry. IEEE Trans. Biomed. Eng. 59, 920–928. doi: 10.1109/TBME.2011.2172210 22010143

[B6] Blankertz B. Tomioka R. Lemm S. Kawanabe M. Müller K.-R. (2008). Optimizing spatial filters for robust EEG single-trial analysis. IEEE Signal Process. Mag. 25, 41–56. doi: 10.1109/MSP.2008.4408441

[B7] Brunner C. Leeb R. Müller-Putz G. Schlögl A. Pfurtscheller G. (2008). BCI Competition 2008-Graz data set A. Graz: Institute for Knowledge Discovery (Laboratory of Brain-Computer Interfaces), Graz University of Technology, 16:1–6.

[B8] Han C. Liu C. Wang J. Wang Y. Cai C. Qian D. (2025). A spatial-spectral and temporal dual prototype network for motor imagery brain-computer interface. Knowl.-Based Syst. 315:113315. doi: 10.1016/j.knosys.2025.113315

[B9] HuJ. ShenL. SunG. (2018). “Squeeze-and-excitation networks,” in Proceedings of the IEEE Conference on Computer Vision and Pattern Recognition (CVPR) (Salt Lake City, UT : IEEE), 7132–7141.

[B10] Jia H. Feng F. Caiafa C. F. Duan F. Zhang Y. Sun Z. . (2023). Multi-class classification of upper limb movements with filter bank task-related component analysis. IEEE J. Biomed. Health Inform. 27, 3867–3877. doi: 10.1109/JBHI.2023.3278747 37227915

[B11] Jia H. Han S. Caiafa C. F. Duan F. Zhang Y. Sun Z. . (2024). Enabling temporal-spectral decoding in multi-class single-side upper limb classification. Eng. Appl. Artif. Intell. 133:108473. doi: 10.1016/j.engappai.2024.108473

[B12] Jiang X. Meng L. Chen X. Xu Y. Wu D. (2024). CSP-Net: Common spatial pattern empowered neural networks for EEG-based motor imagery classification. Knowl.-Based Syst. 305:112668. doi: 10.1016/j.knosys.2024.112668

[B13] Ju C. Guan C. (2023). Graph neural networks on SPD manifolds for motor imagery classification: a perspective from the time-frequency analysis. IEEE Trans. Neural Netw. Learn. Syst. 35, 17405–17419. doi: 10.1109/TNNLS.2023.3307470 37725740

[B14] Koles Z. J. Lazar M. S. Zhou S. Z. (1990). Spatial patterns underlying population differences in the background EEG. Brain Topogr. 2, 275–284. doi: 10.1007/BF01129656 2223384

[B15] Lawhern V. J. Solon A. J. Waytowich N. R. Gordon S. M. Hung C. P. Lance B. J. (2018). EEGNet: a compact convolutional neural network for EEG-based brain-computer interfaces. J. Neural Eng. 15:056013. doi: 10.1088/1741-2552/aace8c 29932424

[B16] Lotte F. Bougrain L. Cichocki A. Clerc M. Congedo M. Rakotomamonjy A. . (2018). A review of classification algorithms for EEG-based brain-computer interfaces: a 10 year update. J. Neural Eng. 15:031005. doi: 10.1088/1741-2552/aab2f2 29488902

[B17] Lotte F. Guan C. (2011). Regularizing common spatial patterns to improve BCI designs: unified theory and new algorithms. IEEE Trans. Biomed. Eng. 58, 355–362. doi: 10.1109/TBME.2010.2082539 20889426

[B18] Mane R. Chew E. Chua K. Ang K. K. Robinson N. Vinod A. P. . (2021). FBCNet: A multi-view convolutional neural network for brain-computer interface. arXiv [preprint] arXiv:2104.01233. doi: 10.48550/arXiv.2104.01233

[B19] Miao Z. Zhao M. Zhang X. Ming D. (2023). Lmda-net: A lightweight multi-dimensional attention network for general eeg-based brain-computer interfaces and interpretability. Neuroimage 276:120209. doi: 10.1016/j.neuroimage.2023.120209 37269957

[B20] MillerK. J. SchalkG. FetzE. E. den NijsM. OjemannJ. G. RaoR. P. (2010). Cortical activity during motor execution, motor imagery, and imagery-based online feedback. Proc. Nat. Acad. Sci. 107, 4430–4435. doi: 10.1073/pnas.091369710720160084 PMC2840149

[B21] Neuper C. Wörtz M. Pfurtscheller G. (2006). ERD/ERS patterns reflecting sensorimotor activation and deactivation. Prog. Brain Res. 159, 211–222. doi: 10.1016/S0079-6123(06)59014-4 17071233

[B22] Park Y. Chung W. (2019). “Optimal channel selection using covariance matrix and cross-combining region in EEG-based BCI,” in Proceedings of the 41st Annual International Conference of the IEEE Engineering in Medicine and Biology Society (EMBC) (Gangwon: IEEE), 4032–4035.

[B23] Pfurtscheller G. Lopes da Silva F. H. (1999). Event-related EEG/MEG synchronization and desynchronization: basic principles. Clini. Neurophysiol. 110, 1842–1857. doi: 10.1016/S1388-2457(99)00141-8 10576479

[B24] Pfurtscheller G. Neuper C. (2001). Motor imagery and direct brain-computer communication. Proc. IEEE 89, 1123–1134. doi: 10.1109/5.939829

[B25] Ramoser H. Müller-Gerking J. Pfurtscheller G. (2000). Optimal spatial filtering of single trial EEG during imagined hand movement. IEEE Trans. Rehabilitat. Eng. 8, 441–446. doi: 10.1109/86.895946 11204034

[B26] SchirrmeisterR. T. SpringenbergJ. T. FiedererL. D. J. GlasstetterM. EggenspergerK. TangermannM. . (2017a). Deep learning with convolutional neural networks for EEG decoding and visualization. Hum. Brain Mapp. 38, 5391–5420. doi: 10.1002/hbm.2373028782865 PMC5655781

[B27] SchirrmeisterR. T. SpringenbergJ. T. FiedererL. D. J. GlasstetterM. EggenspergerK. TangermannM. . (2017b). Deep learning with convolutional neural networks for eeg decoding and visualization. Hum. Brain Mapp. 38, 5391–5420. 28782865 10.1002/hbm.23730PMC5655781

[B28] Subasi A. (2007). EEG signal classification using wavelet feature extraction and a mixture of expert model. Expert Syst. Appl. 32, 1084–1093. doi: 10.1016/j.eswa.2006.02.005

[B29] TangermannM. MüllerK.-R. AertsenA. BirbaumerN. BraunC. BrunnerC. . (2012). Review of the BCI competition IV. Front. Neurosci. 6:55. doi: 10.3389/fnins.2012.0005522811657 PMC3396284

[B30] TongL. QianY. PengL. WangC. HouZ.-G. (2023). A learnable eeg channel selection method for mi-bci using efficient channel attention. Front. Neurosci. 17:1276067. doi: 10.3389/fnins.2023.127606737928726 PMC10622956

[B31] Wimpff M. Gizzi L. Zerfowski J. Yang B. (2024). Eeg motor imagery decoding: a framework for comparative analysis with channel attention mechanisms. J. Neural Eng. 21:036020. doi: 10.1088/1741-2552/ad48b9 38718788

[B32] Wolpaw J. R. Birbaumer N. McFarland D. J. Pfurtscheller G. Vaughan T. M. (2002). Brain-computer interfaces for communication and control. Clini. Neurophysiol. 113, 767–791. doi: 10.1016/S1388-2457(02)00057-3 12048038

[B33] Woo S. Park J. Lee J.-Y. Kweon I. S. (2018). “CBAM: Convolutional block attention module,” in Proceedings of the European Conference on Computer Vision (ECCV), 3–19.

